# Characterization of a Polyacrylamide Solution Used for Remediation of Petroleum Contaminated Soils

**DOI:** 10.3390/ma9010016

**Published:** 2016-01-02

**Authors:** Jongwon Jung, Jungyeon Jang, Jaehun Ahn

**Affiliations:** 1 Department of Civil and Environmental Engineering, Louisiana State University, Baton Rouge, LA 70803, USA; jjung@lsu.edu (J.J.); jjang2@lsu.edu (J.J.); 2 School of Urban, Architecture, and Civil Engineering, Pusan National University, Busan 609-735, Korea

**Keywords:** biopolymer, polyacrylamide, contact angle, surface tension, micromodel, viscosity, soil remediation

## Abstract

Biopolymers are viewed as effective and eco-friendly agents in soil modification. This study focuses on the wettability analysis of polyacrylamide (PAM) solutions for soil remediation. The contact angle, surface tension, and viscosity of PAM solutions were experimentally evaluated in air- and decane-biopolymer solution systems. Furthermore, a micromodel was used to investigate the pore-scale displacement phenomena during the injection of the PAM solution in decane and or air saturated pores. The contact angle of the PAM solution linearly increases with increasing concentration in air but not in decane. The surface tension between the PAM solution and air decreases at increasing concentration. The viscosity of the PAM solution is highly dependent on the concentration of the solution, shear rate, and temperature. Low flow rate and low concentration result in a low displacement ratio level, which is defined as the volume ratio between the injected and the defended fluids in the pores. The displacement ratio is higher for PAM solutions than distilled water; however, a higher concentration does not necessarily guarantees a higher displacement ratio. Soil remediation could be conducted cost-efficiently at high flow rates but with moderate concentration levels.

## 1. Introduction

Recent developments in the use of organic agents for soil improvement have elicited promising results. Organic agents, including polymers, biopolymers, and surfactants, have shown to improve the shear strength, stiffness, soil remediation, and erosion resistance behaviors of geomaterials [1,2,3,4,5,6,7,8]. Among them, biopolymers such as polyacrylamide (PAM) and Xanthan gum have shown great promise for enhanced oil recovery (EOR) because they lead to an increase in the viscosity of water, decrease in the mobility of water, and contact with a larger volume of the reservoir [9,10,11]. Given the water flooding performance of biopolymer solutions through porous media in EOR, they have been considered as cost-efficient materials for oil contaminant soil remediation [12]. However, previous works on biopolymers were limited to the evaluation of the shear strength, stiffness, and erosion resistance properties of soils saturated with biopolymer solutions. Little is known about the flow characteristics of biopolymer solutions through a porous medium. Therefore, in this study, we selected PAM—one of the most widely used biopolymers—and explored its physical properties such as contact angle, surface tension, and viscosity at different concentrations. Furthermore, we conducted micromodel tests to understand the flow behavior of a PAM solution in porous media.

## 2. Literature Review

### 2.1. Remediation of Petroleum Contaminated Soil

Soil contamination in urban and rural environments can be caused at industrial sites such as mining heaps, dumps, filled natural depressions, and quarries [13]. Important characterization and remediation techniques have been developed to deal with the contamination of soils and sediments [14,15]. Soil remediation technologies such as excavation, soil vapor extraction, bioremediation, surfactants enhanced remediation, and steam injection have been used for many years [16,17,18]. The excavation of contaminated soil is a simple solution. However, this method has become less popular owing to its high cost and the lack of available landfill sites [15]. Instead, soil flushing methods such as soil vapor extraction, surfactant-enhanced remediation, and steam injection have been more popular in recent years [19]. Among these, surfactant-enhanced remediation affords an enhanced rate of remediation by exploiting the low surface tension of the surfactants [15]. Oil-contaminated sites have been remediated by in-situ flushing using biosurfactants, that is, surface-active substances synthesized by living cells [20,21]. Biopolymers synthesized from plants can also be used instead of biosurfactants as an eco-friendly soil remediation method [22]. Biopolymer flushing was originally developed for petroleum-enhanced oil recovery, and subsequently, it has been used for the remediation of petroleum waste at contaminated sites [23,24,25,26,27,28,29]. The basic principle of biopolymer flushing is that the addition of a biopolymer to the flushing water leads to increased viscosity and capillary number, decreased mobility, and contact with a larger volume of the reservoir [30]. The capillary number of a fluid is related to the fluid velocity, fluid viscosity, and surface tension. Mobility is a relative measure of how easily a fluid moves through porous media. Apparent mobility is defined as the ratio of the effective permeability to the fluid viscosity [31]. The physical properties of biopolymers, such as viscosity, surface tension, and contact angle, are important to determine when using biopolymer solutions is feasible for the remediation of petroleum contaminated soils.

### 2.2. Multiphase Fluid Flow

**Figure 1:**
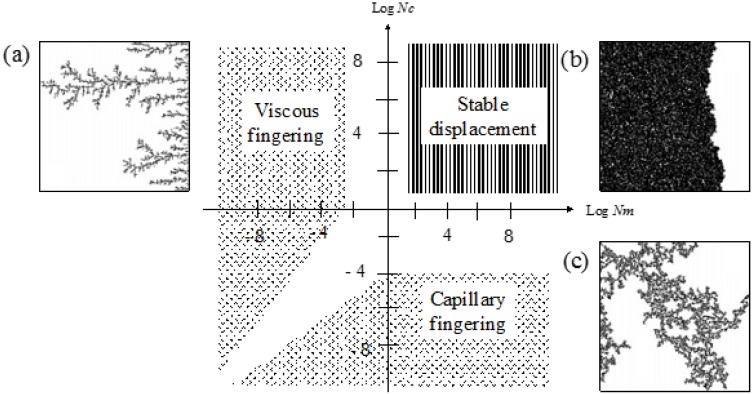
Displacement pattern with respect to *N_m_* and *N_c_*: (**a**) viscous fingering; (**b**) stable displacement; and (**c**) capillary fingering [32,46].

In multiphase fluid flow, the displacement ratio in a porous medium, defined as the ratio of the volume of the injected fluid to that of the defended fluid, is governed by two dimensionless numbers, viscosity number (*N_m_*) and capillary number (*N_c_*), that are defined as follows [32,33]:

(1) $$N_{m}=\frac{\mu_{\text{inv}}}{\mu_{\text{def}}}$$

(2) $$N_{c}=\frac{\mu_{\text{inv}}v_{\text{inv}}}{\sigma \cdot \cos\theta }$$

where *μ*_inv_ and *μ*_def_ are the viscosities of the injected and defended fluids, respectively; *v*_inv_ is the velocity of the injected fluid; *σ* is the surface tension between the injected and the defended fluids; and *θ* is the contact angle on the surface of the medium. These two dimensionless numbers govern the displacement characteristics of the fluids represented by three dominant patterns, capillary fingering, viscous fingering, and stable displacement, as shown in Figure 1 [34,35,36]. When the values of log (*N_m_*) and log (*N_c_*) are positive during fluid injection into a saturated porous medium, stable displacement can be expected. Otherwise, either viscous or capillary fingering may occur, resulting in a low displacement ratio; thus, the volume of petroleum eliminated from the contaminated soil will decrease.

In the experimental program presented in this paper, PAM solution was used as the injected fluid and air or decane was used as the defended fluid. Decane (alkane hydrocarbon, anhydrous, ≥99%, C_10_H_22_) [37,38,39,40,41,42], a major constituent of petroleum, represents the petroleum contaminant in soils [43,44,45].

## 3. Experimental Program

**Figure 2:**
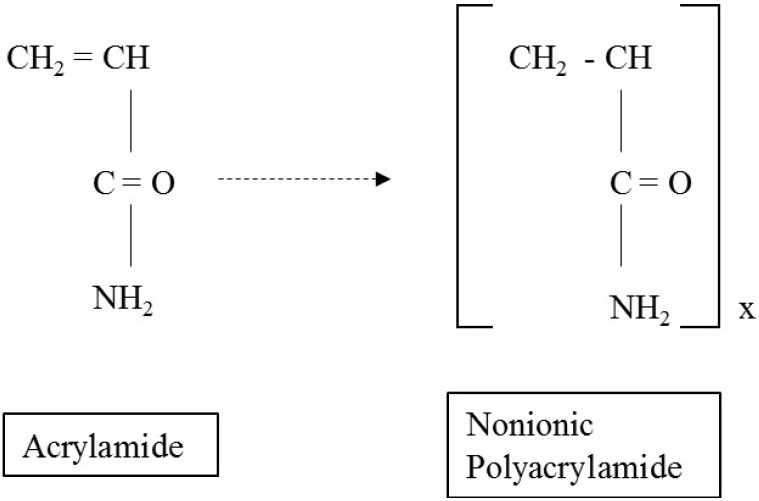
The basic chemical structure of polyacrylamide (PAM) [47].

Figure 2 and Table 1, respectively, show the chemical structure and composition of PAM, a polymer produced using acrylamide. The properties of PAM solutions, such as contact angle, surface tension, viscosity, and flow characteristic (micromodel test), were tested at six concentrations—0, 2, 5, 10, 15, and 20 g/L—prepared by mixing PAM with deionized water, except the viscosity at 0-g/L concentration. The viscosity of deionized water is readily available in literatures.

**Table 1:** The chemical composition of polyacrylamide (PAM).

Label	Name	Chemical Composition
PAM	Polyacrylamide	(C_3_H_5_NO)_n_

### 3.1. Contact Angle

**Figure 3:**
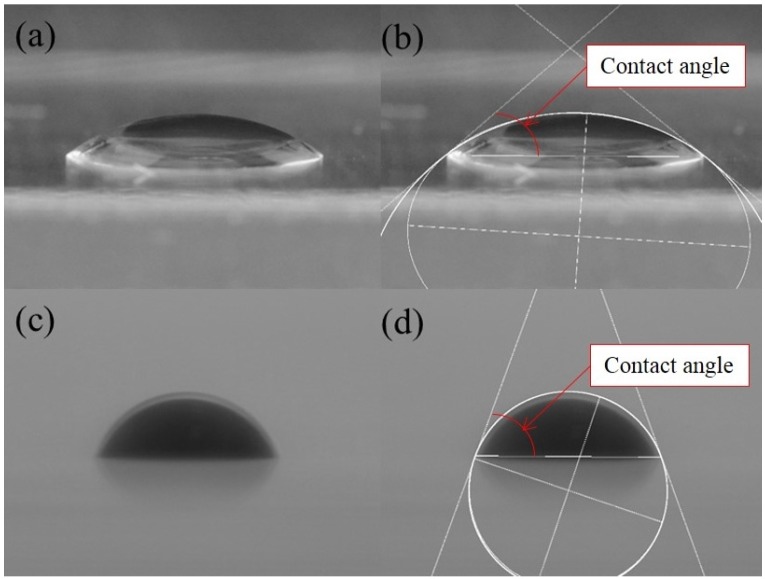
Contact angle on a silica surface (**a**,**b**) in air and (**c**,**d**) in decane as determined using high-resolution time-lapse photography and Image J.

The sessile drop technique was used to measure the contact angle of PAM solutions on the silica surface in air or in decane. Smooth fused pure silica plates (VWR Vista Vision—Cover Glasses, amorphous SiO_2_) were used as the substrates to represent silica sands. Before each contact angle measurement, the silica plate was cleaned using ethanol (Malickrodt Baker, ACS reagent grade) and handled with clean gloves. A new silica plate was used for each measurement to avoid possible complications introduced from the reaction history with decane and PAM solutions. A droplet of PAM solution was foamed on the silica surface under atmospheric conditions, and the contact angle was measured. Furthermore, the PAM solution was introduced into the decane-filled chamber and released onto the silica plate in the chamber. The evolution of the biopolymer droplet was monitored using high-resolution time-lapse photography (Nikon D90, resolution: 12.3 megapixels). Each experimental case was conducted at least five times, at a constant room temperature (24 °C). The captured images of the droplets were analyzed using ImageJ, and the contact angles were measured (Figure 3).

### 3.2. Surface Tension

The surface tension of PAM solutions was measured using the Du Nouy ring method. In this method, a platinum ring of 6-cm diameter is submerged into one phase (liquid) and then raised to another phase to form a fluid meniscus. This meniscus subsequently tears from the ring. The maximum force required to support the ring is measured; this force is the surface tension between the two liquids. In this study, a force tensiometer (Sigma 703D, Biolin Scientific, Stockholm, Sweden) was used for these measurements.

### 3.3. Viscosity

The viscosity of the PAM solutions was measured using a Brookfield Viscometer DV-III (Brookfield, Middleboro, MA, USA). In the tests, a spindle immersed in the test fluid rotates at a constant rate, and the viscosity is measured from the torque required to rotate the spindle. The viscosity of a material can be sensitive to the temperature and loading rate; the experiments were conducted with controlled shear rates (3.4, 6.8, 17, 34, and 68 s^−1^) under various temperatures (25, 50, 70, and 90 °C).

### 3.4. Flow Characteristics (Micromodel Test)

#### 3.4.1. Experimental Setup

**Figure 4:**
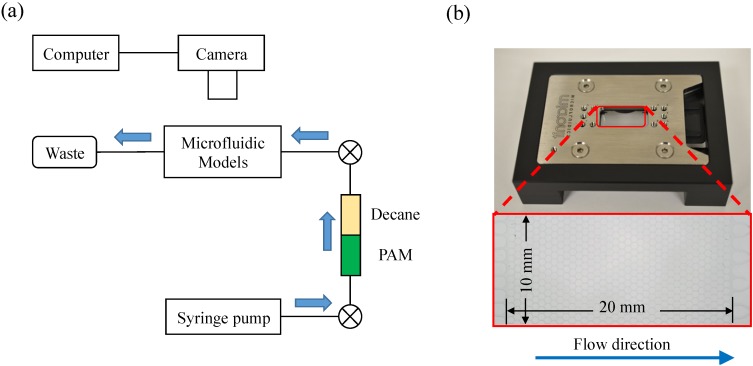
Micromodel test: (**a**) schematic design of the setup and (**b**) micromodel used to represent a two-dimensional porous network.

Figure 4a shows a schematic of the experimental configuration. The silica micromodel was placed horizontally on a customized jack stage. A syringe (2.5 mL with a 1/16 inch fitting) controlled by a precision syringe pump (Kats Scientific, NE-1010, Kats Enterprises, Denton, TX, USA) was connected to the micromodel for injecting the PAM solution. To monitor the flow processes in the micromodel, a high-resolution camera (Nikon D7000, resolution: 16.2 megapixels, Nikon, Tokyo, Japan) was used with computer-controlled image and video capture functions.

The micromodel fabricated by Micronit Microfluidics BV is made out of fused silica. Customized pore networks are etched on two symmetrically patterned silica plates, which are then fused together to form a two-dimensional porous network. The dimension of the patterned area is 20 mm × 10 mm, and it contains 576 discoid silica grains (1186 pore bodies with 590-μm diameter). The pore throat is 20-μm deep and ~30-μm wide. Figure 4b shows the top view of the micromodel within a chip holder for protection and tubing connection.

#### 3.4.2. Experimental Procedure

The micromodel was first cleaned by injecting 5 mL of absolute ethanol (Mallinckodt Baker, ACS reagent grad), and then by 30 mL of deionized water. After cleaning, the micromodel was oven-dried at 120 °C for 48 h. The experimental system was assembled as shown in Figure 4a. Two set of tests were conducted using the micromodel. The first set was the PAM solution injection into the air-saturated micromodel under atmospheric conditions. PAM solution was injected into the micromodel at constant flow rates of 0.3, 1.0, and 50.0 μL/min. The injection was continued until the PAM solution percolated the micromodel, and then additional one hundred pore volume (PV) units of PAM solution, which was sufficient to realize steady displacement, were eventually injected into the micromodel. The second set was the PAM solution injection into the decane-saturated micromodel. A small tubing chamber was placed between the micromodel and the syringe pump that was filled with PAM solution and decane. Decane was always placed at an upper layer in this chamber owing to its lower density compared to the PAM solution, and therefore, decane was pumped first and the micromodel was initially saturated with decane. Then, the PAM solution was injected into the micromodel at constant flow rates of 0.3, 1.0, and 50.0 μL/min. The injection continued until no more decane was drained from the micromodel. Furthermore, additional one hundred PAM solution PV units were eventually injected into the micromodel after the PAM solution had percolated through the micromodel. The room temperature was constant at 24 °C. The images and videos captured during these injection processes were analyzed using ImageJ to compute the biopolymer saturation, or PAM solution-decane displacement ratio, in the micromodel.

## 4. Results

The experimental results for the contact angle, surface tension, viscosity, and flow characteristics (micromodels) are presented and discussed in this section. In addition, the implication of the test results to soil remediation is addressed.

### 4.1. Contact Angle

**Figure 5:**
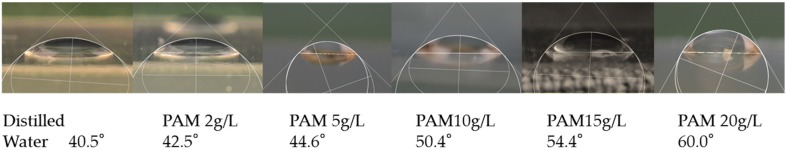
Contact angles at various PAM concentrations on the silica surface in air.

**Figure 6:**
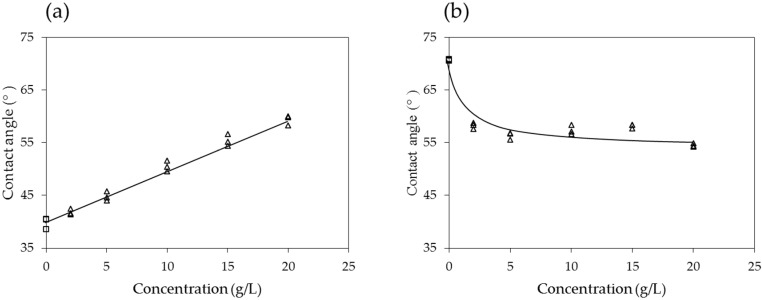
Contact angles of PAM solutions on the silica surface: (**a**) in air and (**b**) in decane.

Figure 5 shows sample snapshots of droplets of PAM solutions and water on the silica plate in air. It can be seen that the contact angles increase as the concentration of the PAM solutions increases. In fact, in air, the contact angle exhibits a linear relationship with the concentration of the PAM solution, as shown in Figure 6a. In decane, however, the contact angles of the PAM solutions are lower than those of water, and the values are relatively constant within the concentration range of 2–20 g/L (Figure 6b). In both air and decane, the contact angles were less than 90°.

### 4.2. Surface Tension

**Figure 7:**
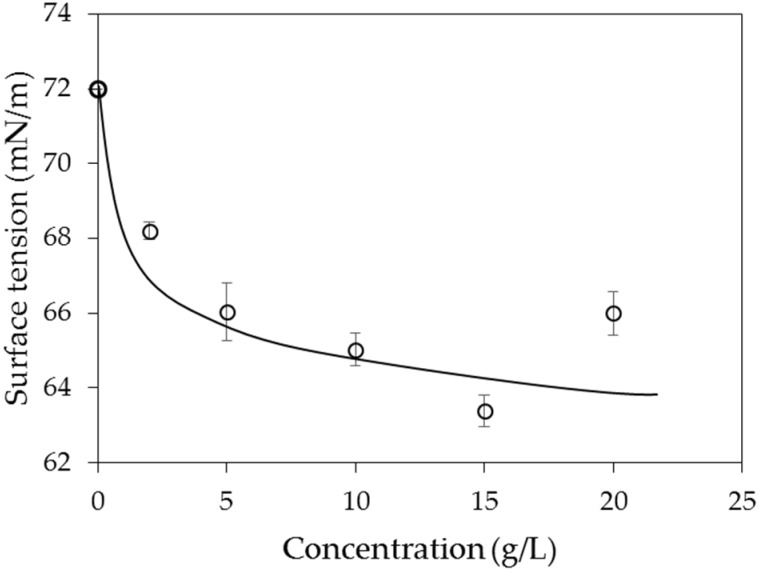
Surface tension of PAM solutions in air.

Figure 7 shows the results of the surface tension of the PAM solutions in air. It can be seen that the PAM solutions have lower surface tension than deionized water, and the value of the surface tension decreases with increasing concentration. The decreasing trend of surface tension is steep up to ~5 g/L, after which it decreases more slowly.

### 4.3. Viscosity

**Figure 8:**
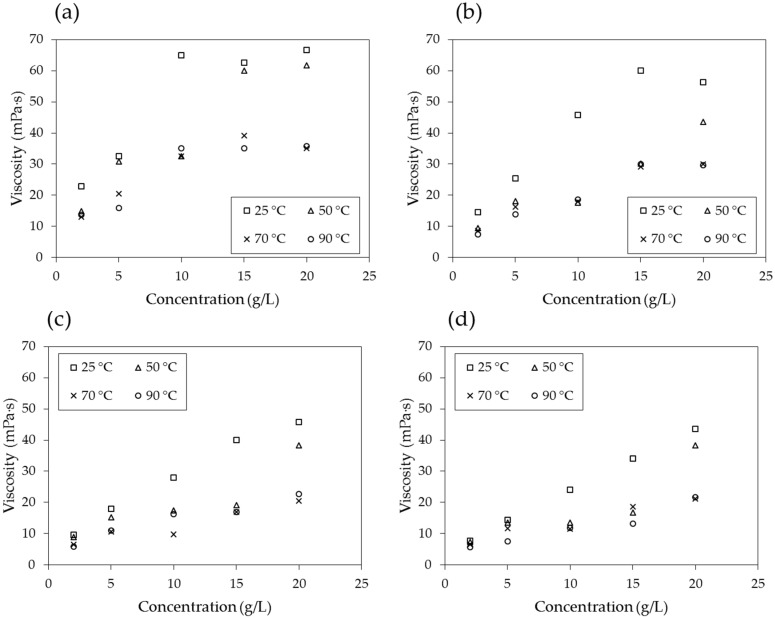

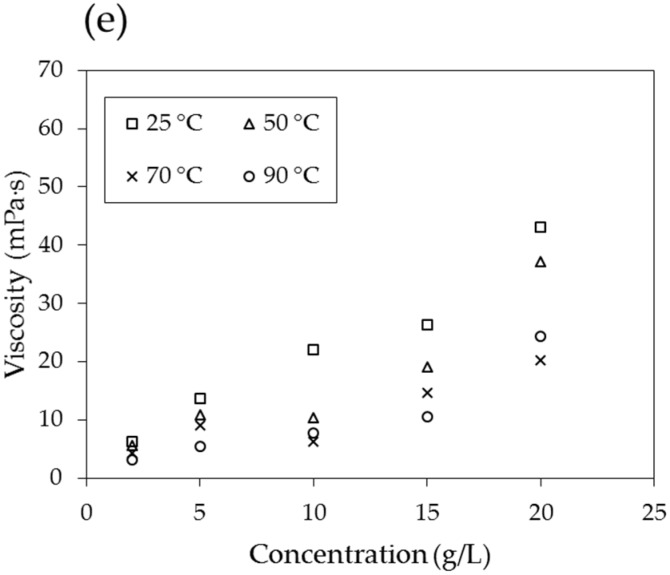
Viscosity with respect to concentration of the solution at shear rates of (**a**) 3.4 s^−1^; (**b**) 6.8 s^−1^; (**c**) 17 s^−1^; (**d**) 34 s^−1^; and (**e**) 68 s^−1^.

Figure 8 shows the results of the viscosities of the PAM solutions with respect to their concentrations at constant shear rates of 3.4, 6.8, 17, 34 and 68 s^−1^. The viscosity tends to increase as the concentration increases, and it has a higher value at a lower temperature and at a higher shear rate. The viscosity consistently increases with increasing concentration at high shear rates of 17–68 s^−1^. However, at lower shear rates of 3.4–6.8 s^−1^, the increasing trend seems less prominent for concentrations higher than 10–15 g/L. 

**Figure 9:**
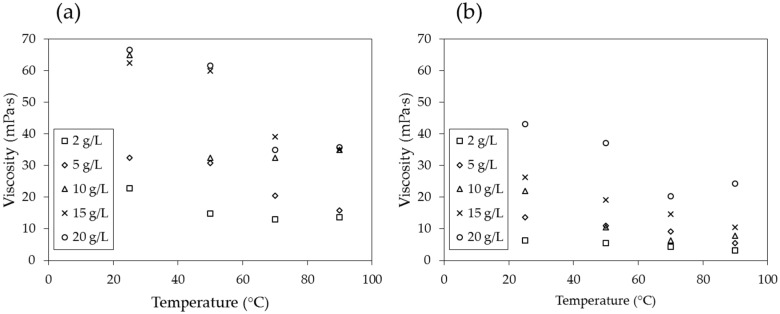
Viscosity with respect to temperature at shear rates of (**a**) 3.4 s^−1^ and (**b**) 68 s^−1^.

Figure 8a,e is regenerated and plotted with respect to temperature in Figure 9a,b, and it becomes clear that the viscosity is inversely proportional to temperature. As the temperature increases, the time of interaction between neighboring molecules of a liquid decreases because of the increased velocities of individual molecules. The intermolecular force decreases, therefore causing the viscosity of the PAM solution to decrease.

**Figure 10:**
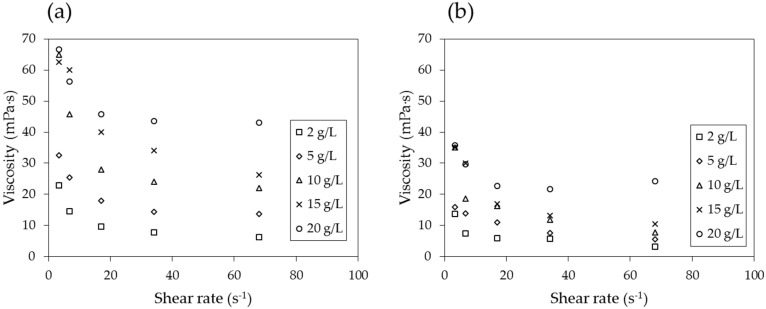
Viscosity with respect to shear rate at temperatures of (**a**) 25 °C and (**b**) 90 °C.

To investigate the effect of the shear rate explicitly, the viscosities are plotted in Figure 10 with respect to the shear rates for the lowest and highest temperatures, 25 and 90 °C. It is noted that the viscosity is dependent on the shear rate, indicating that the biopolymer solution is a non-Newtonian fluid. The viscosity decreases with increasing shear rate up to approximately 20 s^−1^, after which it becomes nearly constant at larger shear rates.

The viscosity of the PAM solution is highly dependent on the concentration of the solution, shear rate, and temperature, as are both *N_m_* and *N_c_*. Therefore, it is important to properly determine the concentration of the PAM solution and shear rate (and, in turn, the injection rate) and estimate the temperature distribution in the ground in order to design the soil remediation process and calculate its efficiency.

### 4.4. Micromodel Tests

**Figure 11:**
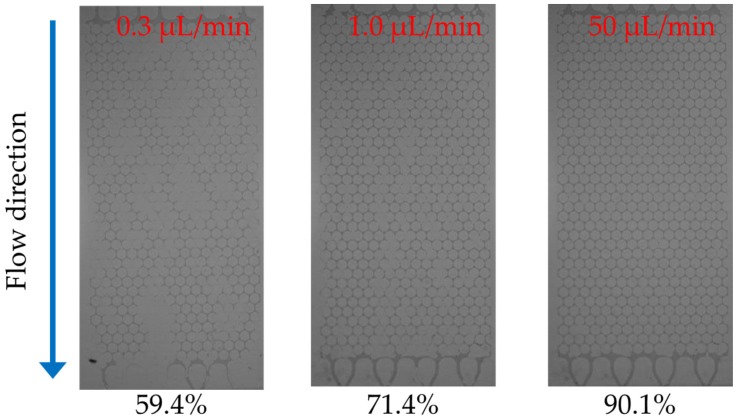
Snapshots of 10-g/L PAM solution injection into the micromodel.

Figure 11 shows a series of snapshots of micromodels upon the injection of 10 g/L PAM solution with additional one hundred times the PV after the first percolation through the micromodel. When these images were analyzed, it was found that the displacement ratio increased at flow rates that ranged from 59.4% at 0.3 μL/min to 90.1% at 50 μL/min. The distribution of the residual decane did not change significantly during the injection of a solution that had hundred times the PV, after the first percolation for all the cases tested.

**Figure 12:**
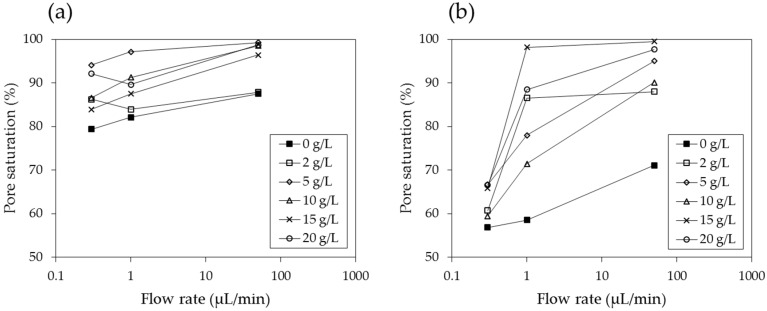
Displacement ratio with respect to flow rate in (**a**) an air saturated model and (**b**) a decane saturated model.

**Figure 13:**
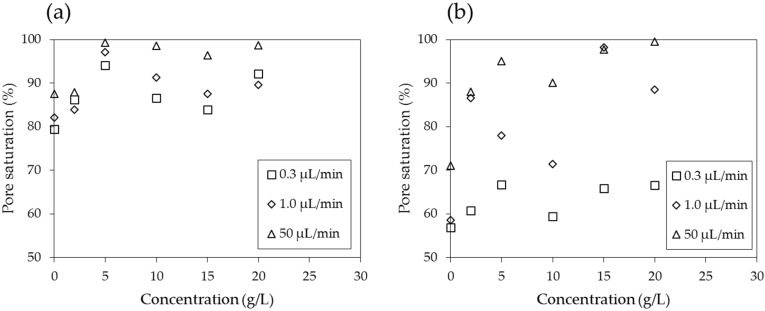
Displacement ratio with respect to concentration in (**a**) an air saturated model and (**b**) a decane saturated model.

Figure 12 shows the values of the displacement ratios with respect to flow rate. While the displacement ratio of the PAM solution against both air and decane consistently increases with increasing values of the flow rate, the effect of the flow rate is more prominent for decane. When the displacement ratio is plotted with respect to the concentration of the solution (Figure 13), for both air and decane saturated systems, the displacement ratio clearly shows an increasing trend for solution concentrations of up to 5 g/L. However, for larger concentrations, the relationship is not straightforward but fluctuates slightly. The results (Figure 12 and Figure 13) show that low flow rates and low concentrations result in low levels of displacement ratios, which is a similar trend to the multiphase flow of CO_2_ and brine, as reported by Kuo *et al*. (2011) [48]. The displacement ratio is higher for PAM solutions than deionized water; however, higher concentrations do not guarantee higher displacement ratios. Soil remediation may be cost efficient at high flow rates but with moderate concentration levels (say, 5 g/L).

### 4.5. Implication to Soil Remediation

When the logarithmic values of the viscosity number (*N_m_*) and capillary number (*N_c_*) both exceed a certain threshold, the flow has stable displacement and the displacement ratio is high (Figure 1). In soil remediation using soil flushing, a higher displacement ratio indicates better remediation of contamination in soils. For an air-PAM solution system, in which the injected fluid is the PAM solution and the defended fluid is air, *N_m_* is higher when the viscosity of the injected PAM solution is higher (see Equation (1)). The PAM solution is a non-Newtonian fluid, as seen in Figure 10. In fact, the viscosity of biopolymer solutions, like that of other polymer solutions, is constant at low shear rate, decreases with increasing shear rate at the moderate level of shear rate, and constant again at high shear rate, whereas the viscosity of air is independent of the shear rate [49]. The viscosity of PAM solution is also sensitive to the temperature and liquid concentration; the viscosity is higher when the temperature is lower (Figure 9), and the concentration is higher (Figure 8). As such, *N_m_* is affected by the injecting rate, and the concentration of PAM solution, and ground temperature. The effect of three controlling values, liquid concentration, injecting rate, and temperature, on *N_c_* in Equation (2) is not as straightforward, though. The liquid concentration itself affects the viscosity of the injecting fluid (Figure 8), surface tension (Figure 7), and contact angle (Figure 6). The injecting rate affects the viscosity and velocity of the injecting fluid (Figure 10), and the temperature influences the viscosity of the injecting fluid (Figure 9). Thus, when designing a soil remediation process, one should be aware that the efficiency of the remediation cannot be enhanced by simply increasing or decreasing some controlling value such as the liquid concentration or injecting rate. The temperature may be more difficult to control; however, the seasonal and spatial variations of the ground temperature are certainly an important factor to consider. The discussion of decane-PAM solutions is more complicated because both phases are non-Newtonian fluids. Further research needs to be conducted to determine better the effect of controlling values and optimal design, including the effect of temperature on the displacement ratio, which is equivalent to the remediation efficiency. Thus far, the obtained results indicate that low injecting rates and low concentrations result in low efficiency, and remediation can be cost-efficient with a high injection rate and moderate concentration levels, as the increase in concentration above 5 g/L does not necessarily guarantee higher displacement.

## 5. Conclusions

A PAM solution was tested for contact angle, surface tension, viscosity, and flow characteristics (micromodels), and the implications to its applications were discussed.

In air, the contact angle of distilled water on a silica surface is ~40°; however, it increases linearly up to ~60° as the concentration of the PAM solution increases to 20 g/L. In decane, the contact angle of distilled water is ~71°; however, it decreases to 54°–59° when PAM is added. The surface tension between the PAM solution and air decreases sharply with increasing concentrations up to ~5 mg/L; however, the rate of decrease subsequently becomes lesser.

The viscosity of the PAM solution is highly dependent on the concentration of the solution, shear rate, and temperature, as are both *N_m_* and *N_c_*. Therefore, it is important to properly determine the concentration of the PAM solution and shear rate (and, in turn, the injection rate) and estimate the temperature distribution in the ground to design the soil remediation process and calculate the efficiency.

The displacement ratio of the PAM solution against air and decane consistently increases with increasing values of flow rates. The effect of the flow rate is more prominent for decane. Low flow rates and low concentrations resulted in low levels of displacement ratios. The displacement ratio is higher for PAM solutions than for deionized water; however, higher concentrations do not guarantee higher displacement ratios. Soil remediation could be conducted cost-efficiently at high flow rates but with moderate concentration levels (~5 g/L).

Further research needs to be conducted to better determine the effect of controlling values and optimal design, including the effect of temperature on the efficiency of remediation.
